# Intertrochanteric Femoral Fractures: A Comparative Analysis of Clinical and Radiographic Outcomes Between Talon Intramedullary Nail and Intertan Nail

**DOI:** 10.7759/cureus.50877

**Published:** 2023-12-21

**Authors:** Hüseyin Kürüm, Hacı Bayram Tosun, Faruk Aydemir, Orhan Ayas, Kübra Orhan, Sefa Key

**Affiliations:** 1 Orthopaedics and Traumatology Department, Ergani State Hospital, Ministry of Health, Diyarbakir, TUR; 2 Orthopaedics Department, Istanbul Medipol University, Istanbul, TUR; 3 Emergency Department, Elazığ Fethi Sekin Training and Research Hospital, Elazığ, TUR; 4 Orthopaedics and Traumatology Department, Elazığ Fethi Sekin Training and Research Hospital, Elazığ, TUR; 5 Physical Therapy and Rehabilitation Department, İnönü University Turgut Özal Medical Center, Malatya, TUR; 6 Orthopedics and Traumatology Department, Firat University Hospital, Firat University, Elazığ, TUR

**Keywords:** evans jansen, elderly hip fractures, intertan pfn, talon pfn, intertrochanteric femur fractures

## Abstract

Introduction

Hip fractures in the elderly constitute a significant health concern, and their incidence is on the rise. It has been reported that intertrochanteric femoral fractures comprise a large portion of hip fractures, and they are especially prevalent among women. Over 75% of these types of fractures in the elderly occur as a result of simple falls. Surgical intervention must be performed for these fractures to expedite the healing process in patients. The application of a proximal femoral nail (PFN) is conducted using a minimally invasive technique after the fracture has been reduced using closed techniques. This technique maintains the fracture hematoma while minimizing the occurrence of consequences such as surgical trauma, hemorrhaging, infection, and issues with the wound site. This study aimed to assess the radiologic and functional outcomes among the groups following surgical procedures utilizing two distinct PFNs.

Methods

Between November 2021 and June 2023, a total of 96 individuals (38 males and 58 females) who underwent surgery for ITF using PFN were included in the study. Our surgical team utilized the Talon™ DistalFix™ PFN system (Orthopedic Designs North America Inc., FL, USA) and the Trigen InterTAN® nail (Smith & Nephew).

Results

The surgery time (number of scopes) for the Talon PFN was recorded as 25 (25-30), while it was 30 (30-35) in the InterTAN group (p<0.001). No nail protrusion was observed in the InterTAN group, whereas nail protrusion was observed in 12 patients (31.6%) in the Talon PFN group (p<0.001). Nail jamming was observed in two (5.3%) patients in the Talon PFN group, while none was observed in the InterTAN group (p<0.07).

Conclusion

In ITF fractures, the InterTAN nail is a more reliable implant. The shorter surgery time, reduced radiation exposure, and more minimally invasive nature of the Talon PFN might be preferred for geriatric patient populations with comorbidities where prolonged anesthesia could elevate mortality risks or for fractures of two or three pieces (Evans-Jansen Type 1 and Type 2). However, for more unstable fractures (Evans-Jensen Type 3) and in the active elderly patient group, we recommend the use of the InterTAN nail.

## Introduction

Hip fractures in the elderly are a pressing health issue, with an increasing incidence [1]. Fractures in the intertrochanteric region are known to constitute a significant portion of hip fractures, which are especially prevalent among women. Over 75% of these fractures in the elderly result from simple falls [2]. Various classification techniques have standardized the types of fractures. Intertrochanteric femur fractures, primarily categorized as unstable or stable, are generally classified under the Evans-Jensen, Boyd and Griffin, and AO/ASIF classifications [3].

A surgical approach is recommended for these fractures to achieve an acceptable reduction and ensure the patient's swift recovery [4]. The use of a PFN is possible through a minimally invasive procedure. By performing a closed reduction of the fracture and preserving the fracture hematoma, the surgeon can undertake a minimally invasive procedure through very small incisions, subsequently reducing blood loss, surgical trauma, infection rates, and wound complications [4-6].

Among the most commonly used intramedullary systems are the proximal femoral nail (PFN), PFN antirotation (PFNA), and Talon, Gamma, and Veronail implant systems. Despite these implant systems having fundamentally similar operational principles, their design features lead to respective advantages and disadvantages. The ultimate goal in all implant systems is to establish a firm interface between the implant and the bone tissue, ensuring and maintaining stability [7,8].

In this study, a total of 96 patients with femoral intertrochanteric fractures were retrospectively compiled, aiming to evaluate the complication rates and clinical and radiological outcomes between the Talon PFN and the InterTAN groups.

## Materials and methods

Between 2021 and 2023, a total of 96 individuals (38 males and 58 females) with ITF were surgically treated and retrospectively analyzed. Our surgical team utilized the Talon™ DistalFix™ Proximal Femoral Nail System (Orthopedic Designs North America Inc., FL, USA) and the Trigen InterTAN® nail (Smith & Nephew). All imaging and medical records were obtained from the hospital's electronic patient record system (Picture Archiving and Communication System-PACS software). The study protocol was approved by the Firat University Hospital Human Subject Research Ethics Committee (2023/12-07). Data collection and evaluation were conducted per the Helsinki Declaration. All patients were informed about the treatment, and their written consents were obtained.

Individuals with unilateral isolated ITF who were mobile enough to perform daily tasks before the injury, at least 18 years old, and had a follow-up period of at least one year participated in the study. Bilateral fractures, pathological fractures, patients with multiple trauma, impaired muscle strength, dementia, and mental disorders such as less than one year of follow-up were the exclusion criteria. 

All patients underwent a standard radiological evaluation that included lateral and anteroposterior (AP) views during the application and follow-up. All fractures were graded according to Evans-Jensen [3].

Patients' age, gender, body mass index (BMI), whether or not they had postoperative intensive care stay, fracture healing times, mortality rate in the first year, surgical or fluoroscopy time, fracture type and reduction quality, tip apex distance (TAD), wound site infection, reduction loss, varus malposition, V-effect, nail jamming, complications like the femur tip protruding, and clinical and radiological results were recorded. At the end of the follow-up period, the Harris Hip Score was used to evaluate hip function, and the results were classified as excellent (90-100), good (80-89), moderate (70-79), and poor (<69).

In the anteroposterior X-ray, the reduction was considered good when there was a normal or slight valgus alignment, angulation <20° in the lateral X-ray, and no displacement of >4 mm in any fragment [9]. The surgeon who was experienced in the treatment and management of hip fractures (HBT) but did not participate in the patient's care reviewed the patient's radiological results, including fracture type, reduction loss, V-effect, varus malposition, type overflow, nail jamming, and other radiological complications. Fracture healing in radiology was characterized by the existence of a bridge callus in a minimum of three out of the four cortices, as observed in anteroposterior and lateral hip radiographs [10]. Nail jamming was reported by the operating surgeon. Loss of reduction was detected as proximal femoral nail (PFN) cutout on postoperative outpatient clinic follow-up X-ray radiography. The term "nail overhang" refers to the section of the nail that extends over the level of the trochanter major on the radiograph performed after surgery. A varus above 5 degrees in the femoral neck region was determined as malposition [11].

All patients were administered with low molecular weight heparin to prevent venous thromboembolism (VTE). All patients began walking as much as they could tolerate with a walker, beginning quadriceps exercises on the first postoperative day. Patients were allowed to begin full weight-bearing in the second postoperative week. The duration of the operation and fluoroscopy time were determined as the number of scans taken between the first incision made following the closed reduction of the fracture and the complete closure of the wound. Bone fracture healing was defined as observing at least three cortices united on the radiograph. All patients underwent clinical and radiographic evaluations on the first day, second week, first month, third month, sixth month, and first year after surgery. The Harris hip score was assessed by a single expert six months after the operation.

Surgical Procedure

All surgical procedures were performed by a single surgeon. Patients were operated in the supine position without the use of a traction table.

InterTAN group: Following closed reduction, an approximately 4 cm incision was made 3-5 cm proximal to the top of the greater trochanter. An InterTAN nail suitable for the medullary space diameter was then placed. It was secured with proximal femur-locking screws. Later, the distal screw was placed with the help of a guide.

Talon PFN group: Following closed reduction, an approximately 4 cm incision was made 3-5 cm proximal to the top of the greater trochanter. A Talon nail suitable for the medullary space diameter was then placed. The Talons distal to the nail were then expanded and viewed under fluoroscopy. Finally, using a guide wire, a lag screw was inserted into the femur neck area, and the Talons were expanded.

Statistics

Statistical analysis was performed using the SPSS program (version 21.0, IBM SPSS Statistics for Windows, Armonk, NY). The normal distribution of the data was evaluated through histograms, Q-Q plots, and the Shapiro-Wilk test. When comparing two groups with normal distribution, independent two-sample t-tests were used, while the Mann-Whitney U test was used for groups with non-normal distribution. Pearson's χ2 test was used for categorical variables. Significance was assessed at p < 0.05 level.

## Results

This study included 96 patients with an average age of 85 (78-89) in the Talon group and 83 (72.75-89.25) in the InterTAN group (p<0.75). Average healing times were three and 3.03±0.49, respectively (p<0.66). Demographic features of the groups are given in Table 1.

**Table 1:** Baseline characteristics and demographic data Statistically significant, Mann-Whitney-U and Chi-Square Tests (p<0.05). Data are expressed as mean±standard deviation (SD) and n (%). Body mass index (BMİ).

Variable	Talon PFN (n=38)	InterTAN (n=58)	P value
Age (year), mean±SD	85 (78-89)	83 (72.75-89.25)	0.75
Gender (M/F)			
Female n(%)	22 (57.9%)	36 (62.1%)	0.683
Male n(%)	16 (42.1%)	22 (37.9%)	0.542
Extremity side			
Right n(%)	20 (52.6%)	28 (48.3%)	0.67
Left n(%)	18 (47.4%)	30 (51.7%)	0.63
BMİ (kg/m2) mean±SD	32 (28-33)	33 (28.7-33)	0.7
Union time (months) mean±SD	3.01±0.38	3.03±0.49	0.66

The majority of fractures, 40% (2+38), were of Evans-Jensen type 2B (Table 2) (p<0.001).

**Table 2:** Type of fractures according to the Evans-Jensen classification

Type	Total (n=96)	Talon PFN (n=38) n(%)	InterTAN (n=58) n(%)	P value
1A	1	1 (2.6%)	0	<0,001
1B	26	12 (31.6%)	14 (24.1%)	
2A	17	15 (39.5%)	2 (3.4%)	
2B	40	2 (5.3%)	38 (65.5%)	
3	12	8 (21.1%)	4 (6.9%)	

During surgery, an average of 25 (25-30) fluoroscopic images were taken in the Talon group, while 30 (30-35) were taken in the InterTAN group (p<0.001). On the postoperative first day, four (10.5%) patients in the Talon group and 10 (17.24%) in the InterTAN group were admitted to intensive care (p<0.08). Despite more patients in the InterTAN group being admitted to intensive care, this difference was not statistically significant (Table 3).

**Table 3:** Comparison of the results of the treatment methods

Variable	Talon PFN (n=38)	InterTAN (n=58)	P values
Surgical time (number of scopes) mean±SD	25 (25-30)	30 (30-35)	<0.001
İntensive care hospitalization (number) n(%)	4 (10.5%)	10 (17.24%)	0.08
Union time (months) mean±SD	3.01±0.38	3.03±0.49	0.66
Nonunion n(%)	-	-	-
Infection n (%)	0	2 (5.3%)	0.07
The overhang of the femoral tip n(%)	12 (31.6%)	0	<0.001
Nail jamming n(%)	2 (5.3%)	0	0.07
Cutout nail n(%)	1 (2.6%)	0	0.2
TAD (tip apex distance/under 25mm) n(%)	36 (94.7%)	48 (82.8%)	0.08
V-effect n(%)	8 (21.1%)	2 (3.4%)	0.006
Varus malposition n(%)	2 (5.26%)	3 (5.17%)	0.63

**Figure 1:**
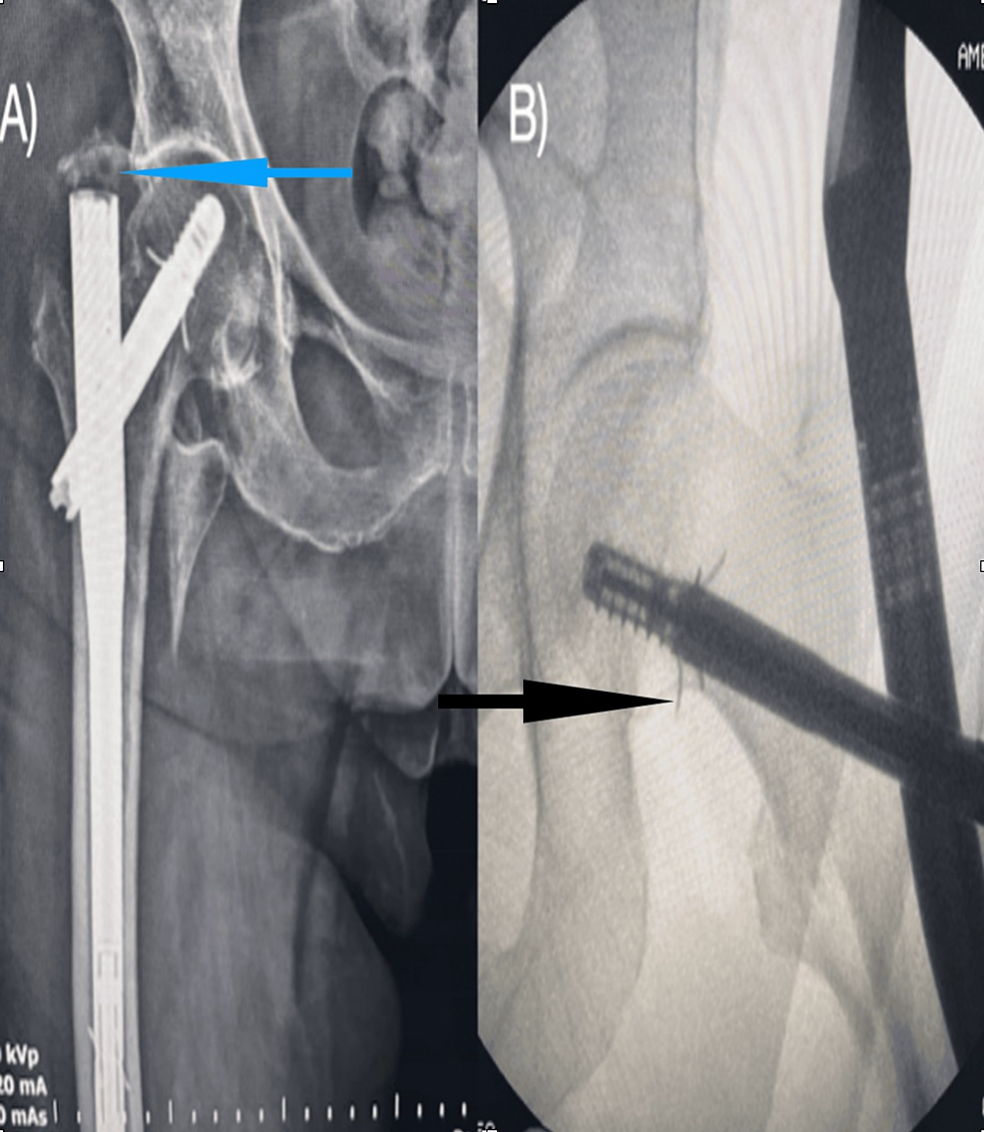
A) Overflow of the Talon PFN from the type because of the lag screw that could not be sent to the lower 1/3 region (blue-arrowed area); B) overflow of the opened wings of the lag screw sent to the femoral neck calcaneal region from the femoral neck (black-arrowed area)

In the Talon group, femur tip overflow was observed in 12 (31.6%) patients, whereas it was not observed in any patients from the InterTAN group (p<0.001) (Figure 1/2). This was manifested as pain in the lateral thigh, and there was a significant difference between the groups (Table 3).

 In the Talon group, nail jamming was observed in two (5.3%) patients, but not in the InterTAN group (p<0.07). TAD was under 25 mm in 36 (94.7%) patients in the Talon group and 48 (82.8%) in the InterTAN group (p<0.08). The V effect was observed in eight (21.8%) patients in the Talon group and two (3.4%) in the InterTAN group (p<0.006). Varus malposition was observed in two (5.26%) patients in the Talon group and three (5.17%) in the InterTAN group (p<0.63) (Table 3).

No significant difference was observed between the two groups in terms of Harris hip scores (Table 4). In the Talon group, 16 (42.1%) were rated good, 12 (31.6%) were moderate, and 10 (26.3%) were poor. In the InterTAN group, 30 (51.7%) were rated good, 13 (22.4%) were moderate, and 15 (25.9%) were rated poor (p<0.553).

**Table 4:** Haris hip score

Scoring	Talon PFN (n=38) n (%)	InterTAN (n=58) n (%)	P value
Excellent (90-100)	-	-	<0,553
Good (80-89)	16 (42.1%)	30 (51.7%)	
Fair (70-79)	12 (31.6%)	13 (22.4%)	
Poor (<69)	10 (26.3%)	15 (25.9%)	

Patients with medical disorders including diabetes, hypertension, chronic renal failure, and heart failure were classified as positive, while those without those diseases were classified as negative. However, the presence or absence of comorbidities among patients in the intensive care unit did not show any statistically significant difference (p<0.67).

## Discussion

In the study, Talon PFN and InterTAN PFN models were compared. Although both models have preferable aspects, they also have disadvantages. The study showed that choosing the implant according to the patient can make these implants more effective.

Talon PFNs have Talon-locking mechanisms both in the femur diaphysis and in the lag screw sent to the femur neck. In a comparison of three different PFN systems, it was reported that Talon-locked PFNs provide fixation as stable as InterTAN and PFNAs and have a much shorter surgical time compared to other systems. The PFN with a Talon-locking system has provided a more practical method for distal femoral fixation [12,13]. Many studies report that distal-locking prolongs the operation time, causes more blood loss, and results in more radiation exposure [14-16]. Stone et al. [17] reported that a significant portion of hip fracture patients is elderly patients with various systemic problems; hence, surgical time is a significant issue in terms of patient prognosis and mortality. In our study, it was observed that the Talon PFN has a shorter surgical time compared to the InterTAN group because its distal locking occurs within the nail only with the help of a screwdriver, without the need for an extra incision. It was observed that fewer patients treated with Talon PFN were admitted to intensive care postoperatively compared to the InterTAN group. In cases where we do not want a long surgery time and expect wound problems in patients with systemic issues, Talon PFN can be primarily preferred.

Studies have shown that the amount of space between the medulla and the nail affects fracture healing. It has been found that nails filling the medulla in femur shaft fractures reduce interfragmentary movement, resulting in better fracture healing. To reduce revision rates, 90% nail fit is required [18,19]. In the Talon PFN group, because the only nail option is 11 mm wide in the distal part, resistance against rotational and axial forces in osteoporotic patients with a broad medulla is provided solely by Talon hooks. In patients with a narrow medulla, the Talons could not be fully expanded, which also caused nail jamming. No screw fracture was observed in any patient in the InterTAN group. Because of the lack of influence from the patient's medulla, the distal-locking system provides a more accurate implant fit in patients who fall within the InterTAN group.

**Figure 2:**
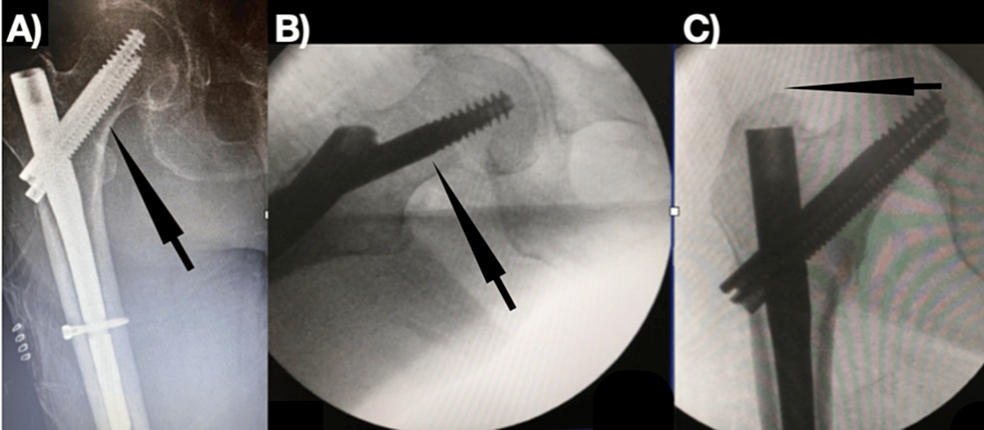
A) The lag screws of the InterTAN nail are placed in the lower third of the femur (the arrow-marked area); B) InterTAN intraoperative femoral neck figured fluoroscopy image (the arrow-marked area); C) InterTAN nail is noted to be flush with the femoral apex, without any protrusion (the arrow-marked area)

In studies, even if TAD <25 mm, it has been shown that lag screws placed in the upper or anterior quadrant can increase the risk of failure with axial loading [20,21]. Yapici et al. [12] demonstrated in their study that the lag screw sent to the femur neck region in the Talon PFN group cut the inferior cortex of the femur. In the Talon PFN group, the lag screw sent to the lower 1/3 region of the femur neck could not be fully opened as its Talon hooks might protrude from the bone cortex, affecting the antirotational effectiveness of the lag screw (Figure 1). In the Talon PFN group, to fully open the Talon hooks of the lag screw, the screw should be placed close to the center of the femur neck. In the InterTAN group, in all patients, the lag screw was sent to the lower 1/3 region of the femur neck along with the compression screw, so there is no decrease in the effectiveness of the nail (Figure 2).

In our study, while the Talon-locked PFN group had 25 (25-30) fluoroscopy counts, this was identified as 30 (30-35) in the InterTAN group (p<0.001). A statistically significant reduction in the number of fluoroscopies and, therefore, radiation exposure, was observed in the Talon PFN group compared to the InterTAN group.

**Figure 3:**
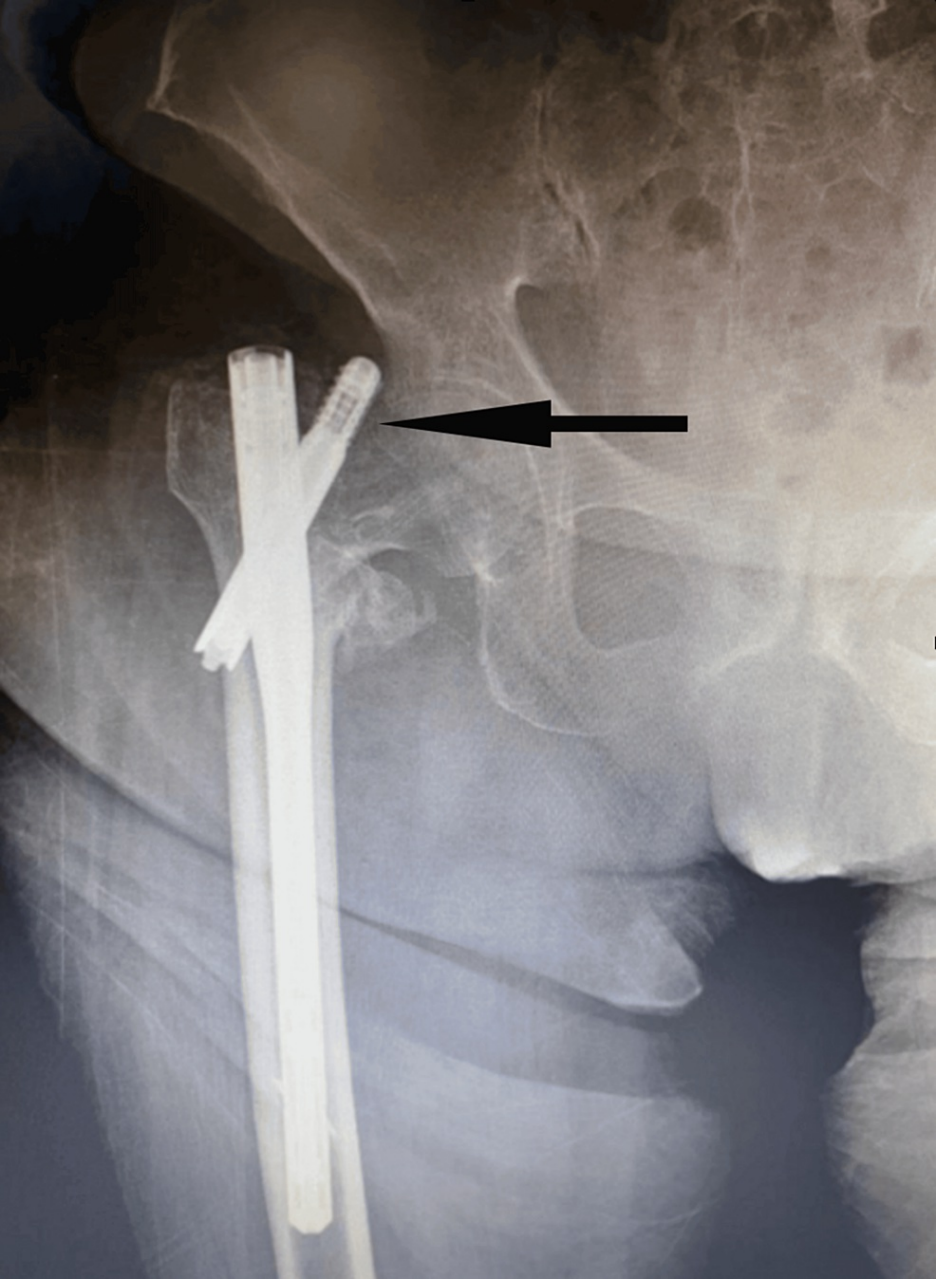
Postoperative 15th day X-ray radiograph of the patient who developed failure because of the failure of the lag screw Talon-lock mechanism (black-arrowed area)

When the nail passes through the major trochanter entry, it slightly damages the gluteus medius muscle, which causes thigh and hip pain in 90.1% of patients [22,23]. In the Talon PFN group, to fully open the Talon hooks of the lag screw, the screw should be placed close to the center of the femur neck. This increases the risk of failure and causes the nail to protrude slightly from the femur tip. In the study, protrusion was observed in 12 (31.6%) patients in the Talon PFN group, while no protrusion was observed in any patient in the InterTAN group (p<0.001). Clinically, this situation causes hip and thigh pain in patients. The study observed failure in one patient, but this was mostly because of the malfunction of the locking mechanism of the lag screw's Talons (Figure 3).

Many studies have reported the postoperative outcomes of patients treated using different implant types related to PFNs. Integrated PFNs, which send a compression lag screw proximally and then lock, have been observed to show less varus collapse and maintain reduction better than PFNs using a single lag screw proximally [24]. In patients treated with the InterTAN nail, the risk of varus collapse, screw migration, and femoral shaft fracture has significantly decreased [25]. In the study, varus was observed in three (5.17%) patients in the InterTAN group and two (5.26%) patients in the Talon group. The occurrence of varus collapse is more related to the quality and sustainability of the reduction than the type of implant used.

Ruecker et al. [26] reported in their study that repeated drilling while sending the distal-locking screw can weaken the lateral cortex, leading to femoral shaft fractures. Similarly, in the InterTAN group, the system did not receive the distal-locking screw in one patient, and it was sent freely. As a result, a second drilling process was applied, and the femur's lateral cortex became weaker. This situation led to an extended surgery time and fluoroscopy duration; however, it did not cause a femoral shaft fracture. The reason for this is the wear of the long-used nail set, preventing it from receiving the distal screw. To prevent this situation, worn sets should be identified preoperatively and revised.

The limitations of this study are the low number of patients, its retrospective design, the use of two different intramedullary nails, and the presence of different fracture types.

## Conclusions

In ITF fractures, the InterTAN nail is a more reliable implant. Talon PFN, with its shorter surgical duration, reduced radiation exposure, and more minimally invasive nature, may be preferred in the geriatric patient population with additional diseases, where prolonged anesthesia may increase the risk of mortality, or in two- or three-piece fracture types (Evans-Jensen Type 1 and Type 2). However, for more unstable fractures (Evans-Jensen Type 3) and the active elderly patient group, we recommend the use of the InterTAN nail.
